# Supplementary figures and images for: Sex-specific differences in symbiotic microorganisms associated with an invasive mealybug (Phenacoccus solenopsis Tinsley) based on 16S ribosomal DNA
Source: PeerJ. 2023 Aug 14;11:e15843. doi: 10.7717/peerj.15843 (PMC10434102; doi:10.7717/peerj.15843)

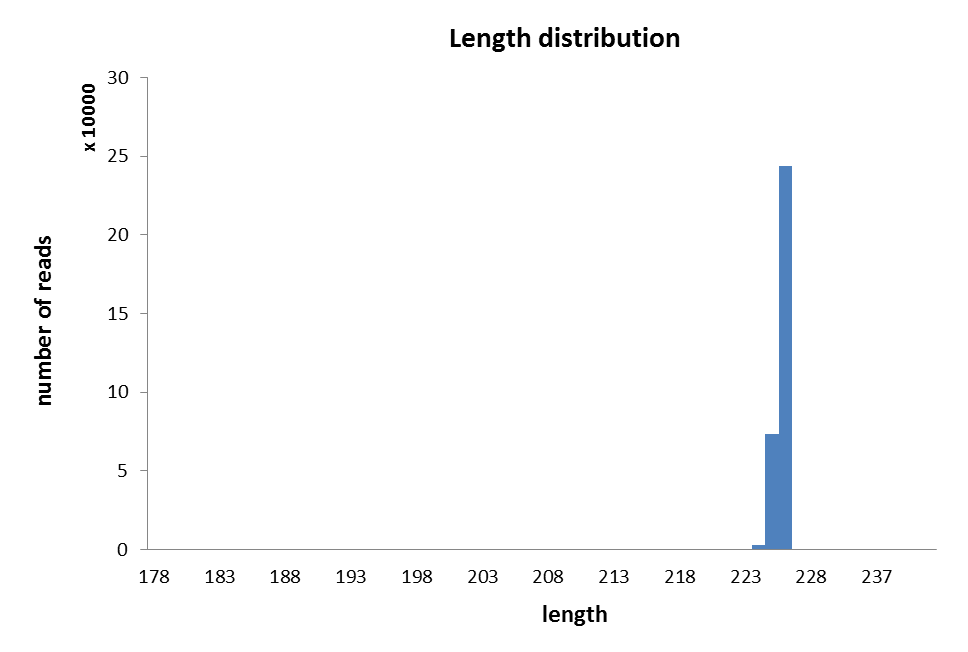

Supplement: Supplemental Information 1 [file peerj-11-15843-s001.png]
