# Supplementary material for: Sex-specific differences in symbiotic microorganisms associated with an invasive mealybug (Phenacoccus solenopsis Tinsley) based on 16S ribosomal DNA
Source: PeerJ. 2023 Aug 14;11:e15843. doi: 10.7717/peerj.15843 (PMC10434102; doi:10.7717/peerj.15843)
Supplement: Supplemental Information 2 [file peerj-11-15843-s002.docx]

Table S1 Genus abundance of male and female mealybugs and difference time among them

| Genus | Abundance | | Difference time  log2(M/F) |
| --- | --- | --- | --- |
|  | Male(M) | Female(F) |  |
| Bacteria;Firmicutes;Clostridia;Clostridiales;Ruminococcaceae;Acetivibrio | 0.001 | 5.271793926 | -12.36407826 |
| Bacteria;Proteobacteria;Betaproteobacteria;Burkholderiales;Alcaligenaceae;Achromobacter | 18.73426614 | 0.001 | 14.19339185 |
| Bacteria;Proteobacteria;Betaproteobacteria;Burkholderiales;Comamonadaceae;Acidovorax | 2.341783268 | 2.635896963 | -0.170686416 |
| Bacteria;Proteobacteria;Gammaproteobacteria;Pseudomonadales;Moraxellaceae;Acinetobacter | 114632.6327 | 13295.46428 | 3.108011736 |
| Bacteria;Actinobacteria;Actinobacteria;Actinomycetales;Actinomycetaceae;Actinomyces | 39.81031556 | 5.271793926 | 2.916776425 |
| Bacteria;Actinobacteria;Actinobacteria;Bifidobacteriales;Bifidobacteriaceae;Aeriscardovia | 16.39248288 | 7.907690888 | 1.051706005 |
| Bacteria;Firmicutes;Bacilli;Lactobacillales;Aerococcaceae;Aerococcus | 203.7351443 | 23.72307267 | 3.102332078 |
| Bacteria;Actinobacteria;Actinobacteria;Actinomycetales;Nocardioidaceae;Aeromicrobium | 9.367133072 | 0.001 | 13.19339185 |
| Bacteria;Verrucomicrobia;Verrucomicrobiae;Verrucomicrobiales;Verrucomicrobiaceae;Akkermansia | 2.341783268 | 36.90255748 | -3.978041338 |
| Bacteria;Proteobacteria;Gammaproteobacteria;Oceanospirillales;Alcanivoracaceae;Alcanivorax | 9.367133072 | 0.001 | 13.19339185 |
| Bacteria;Bacteroidetes;Bacteroidia;Bacteroidales;Rikenellaceae;Alistipes | 42.15209882 | 18.45127874 | 1.191883663 |
| Bacteria;Firmicutes;Erysipelotrichi;Erysipelotrichales;Erysipelotrichaceae;Allobaculum | 14.05069961 | 0.001 | 13.77835435 |
| Bacteria;Proteobacteria;Alphaproteobacteria;Rhizobiales;Phyllobacteriaceae;Aminobacter | 533.9265851 | 92.2563937 | 2.532920581 |
| Bacteria;Firmicutes;Clostridia;Clostridiales;Incertae Sedis XI;Anaerococcus | 9.367133072 | 0.001 | 13.19339185 |
| Bacteria;Tenericutes;Mollicutes;Anaeroplasmatales;Anaeroplasmataceae;Anaeroplasma | 0.001 | 5.271793926 | -12.36407826 |
| Bacteria;Firmicutes;Clostridia;Clostridiales;Lachnospiraceae;Anaerosporobacter | 0.001 | 2.635896963 | -11.36407826 |
| Bacteria;Firmicutes;Bacilli;Bacillales;Paenibacillaceae;Aneurinibacillus | 16.39248288 | 0.001 | 14.00074677 |
| Bacteria;Firmicutes;Bacilli;Bacillales;Bacillaceae;Anoxybacillus | 234.1783268 | 5.271793926 | 5.473169774 |
| Bacteria;Firmicutes;Bacilli;Lactobacillales;Carnobacteriaceae;Atopostipes | 25.75961595 | 0.001 | 14.65282346 |
| Bacteria;Proteobacteria;Gammaproteobacteria;Pseudomonadales;Pseudomonadaceae;Azomonas | 2.341783268 | 0.001 | 11.19339185 |
| Bacteria;Proteobacteria;Alphaproteobacteria;Rhodospirillales;Rhodospirillaceae;Azospirillum | 0.001 | 21.0871757 | -14.36407826 |
| Bacteria;Firmicutes;Bacilli;Bacillales;Bacillaceae;Bacillus | 21.07604941 | 0.001 | 14.36331685 |
| Bacteria;Bacteroidetes;Bacteroidia;Bacteroidales;Bacteroidaceae;Bacteroides | 4.683566536 | 2.635896963 | 0.829313584 |
| Bacteria;Bacteroidetes;Bacteroidia;Bacteroidales;Porphyromonadaceae;Barnesiella | 4.683566536 | 0.001 | 12.19339185 |
| Bacteria;Actinobacteria;Actinobacteria;Bifidobacteriales;Bifidobacteriaceae;Bifidobacterium | 2.341783268 | 10.54358785 | -2.170686416 |
| Bacteria;Firmicutes;Clostridia;Clostridiales;Incertae Sedis XIV;Blautia | 0.001 | 7.907690888 | -12.94904076 |
| Bacteria;Proteobacteria;Alphaproteobacteria;Rhizobiales;Bradyrhizobiaceae;Bosea | 46.83566536 | 23.72307267 | 0.981316678 |
| Bacteria;Actinobacteria;Actinobacteria;Actinomycetales;Dermabacteraceae;Brachybacterium | 11.70891634 | 5.271793926 | 1.151241679 |
| Bacteria;Proteobacteria;Alphaproteobacteria;Rhizobiales;Bradyrhizobiaceae;Bradyrhizobium | 451.9641707 | 121.2512603 | 1.898208665 |
| Bacteria;Firmicutes;Bacilli;Bacillales;Paenibacillaceae;Brevibacillus | 21.07604941 | 0.001 | 14.36331685 |
| Bacteria;Actinobacteria;Actinobacteria;Actinomycetales;Brevibacteriaceae;Brevibacterium | 302.0900416 | 86.98459977 | 1.79614672 |
| Bacteria;Proteobacteria;Alphaproteobacteria;Caulobacterales;Caulobacteraceae;Brevundimonas | 341.9003571 | 121.2512603 | 1.495576187 |
| Bacteria;Firmicutes;Bacilli;Bacillales;Listeriaceae;Brochothrix | 11.70891634 | 0.001 | 13.51531994 |
| Bacteria;Proteobacteria;Alphaproteobacteria;Rhizobiales;Brucellaceae;Brucella | 332.5332241 | 181.8768904 | 0.870536247 |
| Bacteria;Proteobacteria;Gammaproteobacteria;Pseudomonadales;Pseudomonadaceae;Cellvibrio | 11.70891634 | 0.001 | 13.51531994 |
| Bacteria;Proteobacteria;Gammaproteobacteria;Oceanospirillales;Halomonadaceae;Chromohalobacter | 18.73426614 | 0.001 | 14.19339185 |
| Bacteria;Bacteroidetes;Flavobacteria;Flavobacteriales;Flavobacteriaceae;Chryseobacterium | 56.20279843 | 205.5999631 | -1.871126134 |
| Bacteria;Bacteroidetes;Flavobacteria;Flavobacteriales;Flavobacteriaceae;Cloacibacterium | 4.683566536 | 2.635896963 | 0.829313584 |
| Bacteria;Firmicutes;Clostridia;Clostridiales;Clostridiaceae;Clostridium | 4.683566536 | 15.81538178 | -1.755648917 |
| Bacteria;Firmicutes;Bacilli;Bacillales;Paenibacillaceae;Cohnella | 0.001 | 5.271793926 | -12.36407826 |
| Bacteria;Proteobacteria;Betaproteobacteria;Burkholderiales;Comamonadaceae;Comamonas | 7.025349804 | 0.001 | 12.77835435 |
| Bacteria;Firmicutes;Erysipelotrichi;Erysipelotrichales;Erysipelotrichaceae;Coprobacillus | 0.001 | 2.635896963 | -11.36407826 |
| Bacteria;Actinobacteria;Actinobacteria;Actinomycetales;Corynebacteriaceae;Corynebacterium | 114.7473801 | 44.81024837 | 1.356560587 |
| Bacteria;Deinococcus-Thermus;Deinococci;Deinococcales;Deinococcaceae;Deinococcus | 25.75961595 | 57.98973318 | -1.170686416 |
| Bacteria;Actinobacteria;Actinobacteria;Actinomycetales;Dermatophilaceae;Dermatophilus | 11.70891634 | 0.001 | 13.51531994 |
| Bacteria;Firmicutes;Clostridia;Clostridiales;Peptococcaceae;Desulfosporosinus | 0.001 | 21.0871757 | -14.36407826 |
| Bacteria;Proteobacteria;Deltaproteobacteria;Desulfovibrionales;Desulfovibrionaceae;Desulfovibrio | 28.10139922 | 15.81538178 | 0.829313584 |
| Bacteria;Firmicutes;Bacilli;Lactobacillales;Carnobacteriaceae;Dolosigranulum | 18.73426614 | 0.001 | 14.19339185 |
| Bacteria;Firmicutes;Clostridia;Clostridiales;Lachnospiraceae;Dorea | 9.367133072 | 0.001 | 13.19339185 |
| Bacteria;Bacteroidetes;Sphingobacteria;Sphingobacteriales;Cytophagaceae;Dyadobacter | 37.46853229 | 0.001 | 15.19339185 |
| Bacteria;Bacteroidetes;Flavobacteria;Flavobacteriales;Flavobacteriaceae;Elizabethkingia | 2.341783268 | 10.54358785 | -2.170686416 |
| Bacteria;Proteobacteria;Gammaproteobacteria;Pseudomonadales;Moraxellaceae;Enhydrobacter | 49.17744863 | 10.54358785 | 2.221631007 |
| Bacteria;Firmicutes;Bacilli;Lactobacillales;Enterococcaceae;Enterococcus | 0.001 | 2.635896963 | -11.36407826 |
| Bacteria;Firmicutes;Erysipelotrichi;Erysipelotrichales;Erysipelotrichaceae;Erysipelothrix | 14.05069961 | 13.17948481 | 0.09234799 |
| Bacteria;Proteobacteria;Gammaproteobacteria;Enterobacteriales;Enterobacteriaceae;Escherichia/Shigella | 255.2543762 | 519.2717017 | -1.024553911 |
| Bacteria;Firmicutes;Clostridia;Clostridiales;Eubacteriaceae;Eubacterium | 0.001 | 2.635896963 | -11.36407826 |
| Bacteria;Firmicutes;Clostridia;Clostridiales;Ruminococcaceae;Faecalibacterium | 0.001 | 5.271793926 | -12.36407826 |
| Bacteria;Firmicutes;Clostridia;Clostridiales;Incertae Sedis XI;Finegoldia | 7.025349804 | 0.001 | 12.77835435 |
| Bacteria;Bacteroidetes;Sphingobacteria;Sphingobacteriales;Chitinophagaceae;Flavisolibacter | 0.001 | 2.635896963 | -11.36407826 |
| Bacteria;Bacteroidetes;Flavobacteria;Flavobacteriales;Flavobacteriaceae;Flavobacterium | 4.683566536 | 84.34870281 | -4.170686416 |
| Bacteria;Bacteroidetes;Sphingobacteria;Sphingobacteriales;Cytophagaceae;Flectobacillus | 0.001 | 2.635896963 | -11.36407826 |
| Bacteria;Fusobacteria;Fusobacteria;Fusobacteriales;Fusobacteriaceae;Fusobacterium | 18.73426614 | 0.001 | 14.19339185 |
| Bacteria;Proteobacteria;Gammaproteobacteria;Pasteurellales;Pasteurellaceae;Gallibacterium | 0.001 | 2.635896963 | -11.36407826 |
| Bacteria;Actinobacteria;Actinobacteria;Actinomycetales;Nocardiaceae;Gordonia | 2.341783268 | 0.001 | 11.19339185 |
| Bacteria;Proteobacteria;Gammaproteobacteria;Alteromonadales;Alteromonadaceae;Haliea | 0.001 | 5.271793926 | -12.36407826 |
| Bacteria;Bacteroidetes;Bacteroidia;Bacteroidales;Prevotellaceae;Hallella | 0.001 | 2.635896963 | -11.36407826 |
| Bacteria;Proteobacteria;Gammaproteobacteria;Oceanospirillales;Halomonadaceae;Halomonas | 2.341783268 | 0.001 | 11.19339185 |
| Bacteria;Proteobacteria;Epsilonproteobacteria;Campylobacterales;Helicobacteraceae;Helicobacter | 53.86101516 | 44.81024837 | 0.265412699 |
| Bacteria;Firmicutes;Erysipelotrichi;Erysipelotrichales;Erysipelotrichaceae;Holdemania | 2.341783268 | 0.001 | 11.19339185 |
| Bacteria;Proteobacteria;Gammaproteobacteria;Xanthomonadales;Sinobacteraceae;Hydrocarboniphaga | 103.0384638 | 5.271793926 | 4.288745203 |
| Bacteria;Proteobacteria;Betaproteobacteria;Burkholderiales;Comamonadaceae;Hydrogenophaga | 14.05069961 | 0.001 | 13.77835435 |
| Bacteria;Actinobacteria;Actinobacteria;Actinomycetales;Intrasporangiaceae;Janibacter | 39.81031556 | 7.907690888 | 2.331813925 |
| Bacteria;Actinobacteria;Actinobacteria;Actinomycetales;Micrococcaceae;Kocuria | 11.70891634 | 5.271793926 | 1.151241679 |
| Bacteria;Actinobacteria;Actinobacteria;Actinomycetales;Dermacoccaceae;Kytococcus | 14.05069961 | 2.635896963 | 2.414276085 |
| Bacteria;Proteobacteria;Alphaproteobacteria;Rhizobiales;Xanthobacteraceae;Labrys | 11.70891634 | 2.635896963 | 2.151241679 |
| Bacteria;Firmicutes;Bacilli;Lactobacillales;Lactobacillaceae;Lactobacillus | 93.67133072 | 76.44101192 | 0.293260684 |
| Bacteria;Firmicutes;Bacilli;Lactobacillales;Streptococcaceae;Lactococcus | 25.75961595 | 0.001 | 14.65282346 |
| Bacteria;Actinobacteria;Actinobacteria;Actinomycetales;Microbacteriaceae;Leucobacter | 7.025349804 | 0.001 | 12.77835435 |
| Bacteria;Proteobacteria;Betaproteobacteria;Burkholderiales;Burkholderiaceae;Limnobacter | 812.598794 | 76.44101192 | 3.410124442 |
| Bacteria;Verrucomicrobia;Verrucomicrobiae;Verrucomicrobiales;Verrucomicrobiaceae;Luteolibacter | 0.001 | 26.35896963 | -14.68600636 |
| Bacteria;Proteobacteria;Betaproteobacteria;Burkholderiales;Oxalobacteraceae;Massilia | 11.70891634 | 2.635896963 | 2.151241679 |
| Bacteria;Firmicutes;Clostridia;Clostridiales;Veillonellaceae;Megamonas | 0.001 | 5.271793926 | -12.36407826 |
| Bacteria;Firmicutes;Clostridia;Clostridiales;Veillonellaceae;Megasphaera | 0.001 | 5.271793926 | -12.36407826 |
| Bacteria;Proteobacteria;Alphaproteobacteria;Rhizobiales;Phyllobacteriaceae;Mesorhizobium | 2.341783268 | 0.001 | 11.19339185 |
| Archaea;Euryarchaeota;Methanobacteria;Methanobacteriales;Methanobacteriaceae;Methanobrevibacter | 2.341783268 | 0.001 | 11.19339185 |
| Archaea;Euryarchaeota;Methanomicrobia;Methanomicrobiales;Methanocorpusculaceae;Methanocorpusculum | 11.70891634 | 7.907690888 | 0.566279178 |
| Bacteria;Proteobacteria;Alphaproteobacteria;Rhizobiales;Methylobacteriaceae;Methylobacterium | 262.279726 | 44.81024837 | 2.549205665 |
| Bacteria;Proteobacteria;Betaproteobacteria;Methylophilales;Methylophilaceae;Methylophilus | 358.29284 | 31.63076355 | 3.501738926 |
| Bacteria;Actinobacteria;Actinobacteria;Actinomycetales;Micrococcaceae;Micrococcus | 32.78496575 | 0.001 | 15.00074677 |
| Bacteria;Actinobacteria;Actinobacteria;Actinomycetales;Propionibacteriaceae;Microlunatus | 0.001 | 2.635896963 | -11.36407826 |
| Bacteria;Deferribacteres;Deferribacteres;Deferribacterales;Deferribacteraceae;Mucispirillum | 2.341783268 | 13.17948481 | -2.492614511 |
| Bacteria;Actinobacteria;Actinobacteria;Actinomycetales;Mycobacteriaceae;Mycobacterium | 4.683566536 | 2.635896963 | 0.829313584 |
| Bacteria;Tenericutes;Mollicutes;Mycoplasmatales;Mycoplasmataceae;Mycoplasma | 2.341783268 | 0.001 | 11.19339185 |
| Bacteria;Proteobacteria;Gammaproteobacteria;Xanthomonadales;Sinobacteraceae;Nevskia | 53.86101516 | 18.45127874 | 1.545520618 |
| Bacteria;Actinobacteria;Actinobacteria;Actinomycetales;Nocardioidaceae;Nocardioides | 28.10139922 | 28.99486659 | -0.045155534 |
| Bacteria;Actinobacteria;Actinobacteria;Actinomycetales;Nocardiopsaceae;Nocardiopsis | 9.367133072 | 0.001 | 13.19339185 |
| Bacteria;Bacteroidetes;Bacteroidia;Bacteroidales;Porphyromonadaceae;Odoribacter | 2.341783268 | 13.17948481 | -2.492614511 |
| Bacteria;Verrucomicrobia;Opitutae;Opitutales;Opitutaceae;Opitutus | 14.05069961 | 0.001 | 13.77835435 |
| Bacteria;Firmicutes;Clostridia;Clostridiales;Ruminococcaceae;Oscillibacter | 42.15209882 | 44.81024837 | -0.088224256 |
| Bacteria;Proteobacteria;Gammaproteobacteria;Enterobacteriales;Enterobacteriaceae;Pantoea | 9.367133072 | 0.001 | 13.19339185 |
| Bacteria;Bacteroidetes;Bacteroidia;Bacteroidales;Porphyromonadaceae;Parabacteroides | 58.5445817 | 28.99486659 | 1.013738155 |
| Bacteria;Proteobacteria;Alphaproteobacteria;Rhodobacterales;Rhodobacteraceae;Paracoccus | 39.81031556 | 13.17948481 | 1.59484833 |
| Bacteria;Bacteroidetes;Sphingobacteria;Sphingobacteriales;Chitinophagaceae;Parasegetibacter | 0.001 | 21.0871757 | -14.36407826 |
| Bacteria;Proteobacteria;Betaproteobacteria;Burkholderiales;Alcaligenaceae;Parasutterella | 37.46853229 | 60.62563014 | -0.694248372 |
| Bacteria;Bacteroidetes;Sphingobacteria;Sphingobacteriales;Sphingobacteriaceae;Pedobacter | 28.10139922 | 34.26666052 | -0.286163633 |
| Bacteria;Proteobacteria;Alphaproteobacteria;Rhizobiales;Hyphomicrobiaceae;Pedomicrobium | 11.70891634 | 0.001 | 13.51531994 |
| Bacteria;Firmicutes;Clostridia;Clostridiales;Incertae Sedis XI;Peptoniphilus | 0.001 | 7.907690888 | -12.94904076 |
| Bacteria;Bacteroidetes;Bacteroidia;Bacteroidales;Porphyromonadaceae;Petrimonas | 42.15209882 | 0.001 | 15.36331685 |
| Bacteria;Proteobacteria;Alphaproteobacteria;Caulobacterales;Caulobacteraceae;Phenylobacterium | 28.10139922 | 0.001 | 14.77835435 |
| Bacteria;Proteobacteria;Alphaproteobacteria;Rhizobiales;Phyllobacteriaceae;Phyllobacterium | 0.001 | 5.271793926 | -12.36407826 |
| Bacteria;Bacteroidetes;Bacteroidia;Bacteroidales;Porphyromonadaceae;Porphyromonas | 2.341783268 | 0.001 | 11.19339185 |
| Bacteria;Bacteroidetes;Bacteroidia;Bacteroidales;Prevotellaceae;Prevotella | 14.05069961 | 5.271793926 | 1.414276085 |
| Bacteria;Actinobacteria;Actinobacteria;Actinomycetales;Propionibacteriaceae;Propionibacterium | 70.25349804 | 10.54358785 | 2.73620418 |
| Bacteria;Proteobacteria;Gammaproteobacteria;Enterobacteriales;Enterobacteriaceae;Proteus | 0.001 | 5.271793926 | -12.36407826 |
| Bacteria;Proteobacteria;Alphaproteobacteria;Rhizobiales;Brucellaceae;Pseudochrobactrum | 0.001 | 50.08204229 | -15.61200577 |
| Bacteria;Proteobacteria;Gammaproteobacteria;Pseudomonadales;Pseudomonadaceae;Pseudomonas | 14331.7136 | 2061.271425 | 2.797604724 |
| Bacteria;Proteobacteria;Betaproteobacteria;Burkholderiales;Comamonadaceae;Pseudorhodoferax | 0.001 | 2.635896963 | -11.36407826 |
| Bacteria;Proteobacteria;Gammaproteobacteria;Xanthomonadales;Xanthomonadaceae;Pseudoxanthomonas | 49.17744863 | 0.001 | 15.58570927 |
| Bacteria;Proteobacteria;Gammaproteobacteria;Pseudomonadales;Moraxellaceae;Psychrobacter | 39.81031556 | 5.271793926 | 2.916776425 |
| Bacteria;Proteobacteria;Alphaproteobacteria;Rhizobiales;Rhizobiaceae;Rhizobium | 121.7727299 | 60.62563014 | 1.006191346 |
| Bacteria;Proteobacteria;Alphaproteobacteria;Rhodobacterales;Rhodobacteraceae;Rhodobacter | 7.025349804 | 0.001 | 12.77835435 |
| Bacteria;Actinobacteria;Actinobacteria;Actinomycetales;Nocardiaceae;Rhodococcus | 88.98776418 | 5.271793926 | 4.077241098 |
| Bacteria;Firmicutes;Clostridia;Clostridiales;Lachnospiraceae;Roseburia | 4.683566536 | 15.81538178 | -1.755648917 |
| Bacteria;Firmicutes;Clostridia;Clostridiales;Ruminococcaceae;Ruminococcus | 39.81031556 | 76.44101192 | -0.94120457 |
| Bacteria;Proteobacteria;Betaproteobacteria;Burkholderiales;Comamonadaceae;Schlegelella | 9.367133072 | 2.635896963 | 1.829313584 |
| Bacteria;Bacteroidetes;Sphingobacteria;Sphingobacteriales;Chitinophagaceae;Sediminibacterium | 11.70891634 | 15.81538178 | -0.433720822 |
| Bacteria;Bacteroidetes;Sphingobacteria;Sphingobacteriales;Sphingobacteriaceae;Sphingobacterium | 7.025349804 | 181.8768904 | -4.694248372 |
| Bacteria;Proteobacteria;Alphaproteobacteria;Sphingomonadales;Sphingomonadaceae;Sphingobium | 18.73426614 | 0.001 | 14.19339185 |
| Bacteria;Proteobacteria;Alphaproteobacteria;Sphingomonadales;Sphingomonadaceae;Sphingomonas | 365.3181898 | 89.62049673 | 2.027252962 |
| Bacteria;Firmicutes;Clostridia;Clostridiales;Ruminococcaceae;Sporobacter | 9.367133072 | 10.54358785 | -0.170686416 |
| Bacteria;Firmicutes;Bacilli;Bacillales;Staphylococcaceae;Staphylococcus | 39.81031556 | 15.81538178 | 1.331813925 |
| Bacteria;Proteobacteria;Gammaproteobacteria;Xanthomonadales;Xanthomonadaceae;Stenotrophomonas | 23.41783268 | 31.63076355 | -0.433720822 |
| Bacteria;Firmicutes;Bacilli;Lactobacillales;Streptococcaceae;Streptococcus | 42.15209882 | 2.635896963 | 3.999238586 |
| Bacteria;Proteobacteria;Betaproteobacteria;Burkholderiales;Alcaligenaceae;Sutterella | 18.73426614 | 7.907690888 | 1.244351083 |
| Bacteria;Firmicutes;Bacilli;Bacillales;Paenibacillaceae;Thermicanus | 18.73426614 | 0.001 | 14.19339185 |
| Bacteria;Firmicutes;Clostridia;Thermoanaerobacterales;Thermoanaerobacteraceae;Thermoanaerobacterium | 23.41783268 | 0.001 | 14.51531994 |
| Bacteria;Proteobacteria;Gammaproteobacteria;Xanthomonadales;Xanthomonadaceae;Thermomonas | 14.05069961 | 0.001 | 13.77835435 |
| Bacteria;Deinococcus-Thermus;Deinococci;Thermales;Thermaceae;Thermus | 35.12674902 | 2.635896963 | 3.73620418 |
| Bacteria;Spirochaetes;Spirochaetes;Spirochaetales;Spirochaetaceae;Treponema | 0.001 | 21.0871757 | -14.36407826 |
| Bacteria;Actinobacteria;Actinobacteria;Actinomycetales;Tsukamurellaceae;Tsukamurella | 11.70891634 | 0.001 | 13.51531994 |
| Bacteria;Firmicutes;Erysipelotrichi;Erysipelotrichales;Erysipelotrichaceae;Turicibacter | 0.001 | 2.635896963 | -11.36407826 |
| Bacteria;Proteobacteria;Betaproteobacteria;Burkholderiales;Comamonadaceae;Variovorax | 11.70891634 | 0.001 | 13.51531994 |
| Bacteria;Lentisphaerae;Lentisphaeria;Victivallales;Victivallaceae;Victivallis | 0.001 | 47.44614533 | -15.53400326 |
| Bacteria;Proteobacteria;Betaproteobacteria;Neisseriales;Neisseriaceae;Vogesella | 16.39248288 | 0.001 | 14.00074677 |
| Bacteria;Firmicutes;Bacilli;Lactobacillales;Leuconostocaceae;Weissella | 18.73426614 | 10.54358785 | 0.829313584 |
| Bacteria;Proteobacteria;Gammaproteobacteria;Xanthomonadales;Xanthomonadaceae;Wohlfahrtiimonas | 16.39248288 | 2.635896963 | 2.636668506 |
