# Supplementary material for: Sex-specific differences in symbiotic microorganisms associated with an invasive mealybug (Phenacoccus solenopsis Tinsley) based on 16S ribosomal DNA
Source: PeerJ. 2023 Aug 14;11:e15843. doi: 10.7717/peerj.15843 (PMC10434102; doi:10.7717/peerj.15843)
Supplement: Supplemental Information 3 [file peerj-11-15843-s003.docx]

**Table S2 content of the bacterial phyla of male and female mealybugs**

| **Phylum** | **Male mealybug** | **Female mealybug** |
| --- | --- | --- |
|  | **percentage** | **percentage** |
| *Acidobacteria* | 0.00% | 0.00% |
| *Actinobacteria* | 0.24% | 0.06% |
| *Bacteroidetes* | 0.29% | 0.33% |
| *Cyanobacteria* | 0.08% | 0.01% |
| *Deferribacteres* | 0.00% | 0.00% |
| *Deinococcus-Thermus* | 0.02% | 0.02% |
| *Euryarchaeota* | 0.00% | 0.00% |
| *Firmicutes* | 0.64% | 2.92% |
| *Fusobacteria* | 0.00% | 0.01% |
| *Lentisphaerae* | 0.00% | 0.01% |
| *Planctomycetes* | 0.00% | 0.00% |
| *Proteobacteria* | 98.71% | 96.59% |
| *Spirochaetes* | 0.00% | 0.02% |
| *Tenericutes* | 0.00% | 0.00% |
| *TM7* | 0.01% | 0.00% |
| *Verrucomicrobia* | 0.00% | 0.04% |
