# Supplementary material for: Sex-specific differences in symbiotic microorganisms associated with an invasive mealybug (Phenacoccus solenopsis Tinsley) based on 16S ribosomal DNA
Source: PeerJ. 2023 Aug 14;11:e15843. doi: 10.7717/peerj.15843 (PMC10434102; doi:10.7717/peerj.15843)
Supplement: Supplemental Information 4 [file peerj-11-15843-s004.docx]

**Table S3 Content of the first 20 genera of male and female mealybugs**

| **Male mealybug** | | **Female mealybug** | |
| --- | --- | --- | --- |
| **Genera** | **percentage** | **genera** | **percentage** |
| *Acinetobacter* | 84.39% | *Acinetobacter* | 71.85% |
| *Pseudomonas* | 9.13% | *Pseudomonas* | 11.14% |
| *Limnobacter* | 0.57% | *Escherichia/Shigella* | 2.81% |
| *Aminobacter* | 0.38% | *Chryseobacterium* | 1.11% |
| *Bradyrhizobium* | 0.32% | *Brucella* | 0.98% |
| *Sphingomonas* | 0.26% | *Sphingobacterium* | 0.98% |
| *Methylophilus* | 0.25% | *Bradyrhizobium* | 0.66% |
| *Brevundimonas* | 0.24% | *Brevundimonas* | 0.66% |
| *Brucella* | 0.24% | *Aminobacter* | 0.50% |
| *Brevibacterium* | 0.22% | *Sphingomonas* | 0.48% |
| *Methylobacterium* | 0.19% | *Brevibacterium* | 0.47% |
| *Escherichia/Shigella* | 0.18% | *Flavobacterium* | 0.46% |
| *Anoxybacillus* | 0.17% | *Lactobacillus* | 0.41% |
| *Aerococcus* | 0.15% | *Limnobacter* | 0.41% |
| *Rhizobium* | 0.09% | *Ruminococcus* | 0.41% |
| *Corynebacterium* | 0.08% | *Parasutterella* | 0.33% |
| *Hydrocarboniphaga* | 0.07% | *Rhizobium* | 0.33% |
| *Lactobacillus* | 0.07% | *Deinococcus* | 0.31% |
| *Rhodococcus* | 0.06% | *Pseudochrobactrum* | 0.27% |
| *Propionibacterium* | 0.05% | *Victivallis* | 0.26% |
